# The Associations Between White Matter Disruptions and Cognitive Decline at the Early Stage of Subcortical Vascular Cognitive Impairment: A Case–Control Study

**DOI:** 10.3389/fnagi.2021.681208

**Published:** 2021-08-02

**Authors:** Yanan Qiao, Xuwen He, Junying Zhang, Ying Liang, Wen Shao, Zhanjun Zhang, Sihang Zhang, Dantao Peng

**Affiliations:** ^1^Department of Neurology, China-Japan Friendship Hospital, Beijing, China; ^2^State Key Laboratory of Cognitive Neuroscience and Learning & IDG/McGovern Institute for Brain Research, Beijing Normal University, Beijing, China; ^3^School of Biomedical Engineering, Capital Medical University, Beijing, China; ^4^Department of Epidemiology and Biostatistics, School of Public Health, Peking University Health Science Center, Beijing, China

**Keywords:** subcortical vascular cognitive impairment, subcortical ischemic vascular disease, diffusion tensor imaging, white matter hyperintensities, cognitive impairment

## Abstract

**Objective:**

Emerging evidence suggests that white matter (WM) disruption is associated with the incidence of subcortical vascular cognitive impairment (SVCI). However, our knowledge regarding this relationship in the early stage of SVCI is limited. We aimed to investigate the associations between WM disruptions and cognitive declines at the early stage of SVCI.

**Method:**

We performed a case–control study, involving 22 cases and 19 controls. The cases were patients at the early stage of SVCI, which was defined as subcortical ischemic vascular disease with normal global cognitive measures (pre-SVCI). The controls were healthy people matched by age, sex, and education years. We assessed the differences in a battery of neuropsychological tests between the two groups, investigated the diffusion changes in 40 WM tracts among the participants *via* an atlas-based segmentation strategy, and compared the differences between the cases and controls by multiple linear regression analysis. We then evaluated the relationships between diffusion indices and cognitive assessment scores by Pearson’s correlation.

**Results:**

The pre-SVCI group exhibited significant differences in the Montreal cognitive assessment (MoCA), Rey–Osterrieth Complex Figure (R-O)-copy, and Trail Making Test (TMT)-B test compared with the controls. Compared with the controls, some long associative and projective bundles, such as the right anterior corona radiata (ACR), the right inferior fronto-occipital fasciculus (IFOF), and the left external capsule (EC), were extensively damaged in cases after Bonferroni correction (*p* < 0.05/40). Damages to specific fibers, such as the right ACR, IFOF, and posterior thalamic radiation (PTR), exhibited significant correlations with declines in MoCA, R-O delay, and the Mini-Mental State Examination (MMSE), respectively, after Bonferroni correction (*p* < 0.05/14).

**Conclusion:**

Long WM tracts, especially those in the right hemisphere, were extensively damaged in the pre-SVCI patients and correlated with declines in executive functions and spatial processing. Patients of pre-SVCI are likely at an ultra-early stage of SVCI, and there is a very high risk of this condition becoming SVCI.

## Introduction

Subcortical ischemic vascular disease (SIVD) is widespread among elderly individuals with asymptomatic lacunes and subcortical white matter (WM) hyperintensities (Carey et al., 2008), which is a homogeneous and the most common subtype of cerebral small vessel disease (CSVD) (Román et al., 2002; Rosenberg et al., 2014). Subcortical vascular cognitive impairment (SVCI) has a relatively insidious onset with gradual cognitive deterioration and a severe prognosis. In contrast to Alzheimer’s disease (AD), SVCI is widely considered a disease that can be prevented and improved (Sachdev et al., 2014). Therefore, early identification is crucial for preventing SIVD from developing into vascular cognitive impairment (VCI) or dementia.

Most previous studies focused on the moderate and severe stages of SVCI, including subcortical vascular mild cognitive impairment (SvMCI) and subcortical vascular dementia (SVaD). They have revealed significant brain abnormalities in SVCI, such as declines in executive function, attention, processing speed, learning and memory, and lower brain perfusion or abnormal resting-state functional connectivities (FCs) in the thalamus, temporal lobe, inferior frontal lobe, and medial prefrontal cortex (Zhang et al., 2013; Reijmer et al., 2016; Sun et al., 2016; van Leijsen et al., 2019). Among them, WM damage is attracting ever-increasing attention because the pathomechanisms of cognitive injury in SIVD seem to be most closely related to diffuse areas of WM damage with neuronal loss, demyelination, and gliosis (D’Souza et al., 2018).

However, clinicians have also encountered many patients with moderate to serious SIVD with normal global cognition measures, such as the Mini-Mental State Examination (MMSE). The diagnostic criteria for vascular cognitive disorders (VASCOG statement) (Sachdev et al., 2014) suggest that vascular brain damage can exist without any evident cognitive impairment, and such asymptomatic individuals may be at an increased risk of future decline. This stage can be referred to as the pre-stage of SVCI (pre-SVCI). Limited studies focused on these pre-SVCI stage patients. Carey et al. (2008) found that although these patients appear “normal” with normal global cognition measures, they already have poorer executive functions and processing speed based on detailed assessments in different cognitive domains. Moreover, these patients have already exhibited extensive areas of microstructural changes in WM fibers and FC of resting-state networks (Liu et al., 2019a,b). However, these studies mostly used the MMSE as the screening scale. Compared with the Montreal cognitive assessment (MoCA), the MMSE lacks sensitivity in detecting executive function mediated by the frontal lobes where SIVD is often the most prevalent (Tullberg et al., 2004; Carey et al., 2008; Dong et al., 2010, 2016). Using the MMSE alone may lead to a false-negative bias when screening cognitively normal people at the pre-SVCI stage. Therefore, the application of the MMSE and MOCA together as screening scales can more accurately distinguish “normal” patients at the pre-SVCI stage.

Diffusion tensor imaging (DTI) is a sensitive and reliable method used to detect early WM alterations (Nitkunan et al., 2008). Recent DTI studies have demonstrated that patients with cognitive impairment exhibit decreased fractional anisotropy (FA) and increased mean diffusivity (MD), and different combinations of changes in axial diffusivity (DA) and radial diffusivity (DR) of WM tracts especially those located in thalamic- and caudate-prefrontal pathways, such as the corpus callosum (CC), external capsule, and superior and anterior thalamic radiations (ATR) (Chen et al., 2015; Reijmer et al., 2016; D’Souza et al., 2018). These WMs are significantly related to the cognitive domains of executive function, attention, and processing speed. However, limited studies using DTI have been performed in pre-SVCI patients and those that have been performed indicated inconsistent results (Liu et al., 2019a,b; Du et al., 2020). While Liu et al. (2019b) thought the pre-SVCI group exhibited widespread damages in whole-brain WM skeletons, Du et al. (2020) demonstrated well-preserved rich-club organization, less nodal strength loss, and disruption of connections shown in the local connections in the preclinical stage of SVCI. Therefore, to identify pre-SVCI patients at the early stage, we used DTI, which can identify changes in WM microstructure, to perform the current study. The early identification of pre-SVCI should contribute to promoting the further longitudinal studies and the early prevention and treatment of cognitive impairment due to CSVD.

In this explorative case–control study, using a battery of neuropsychological tests and DTI, we aimed to assess changes in 40 WM tracts that can mainly contain the key WMs in the brain (Zhang et al., 2014; Chen et al., 2015) and cognitive domains, including memory, spatial processing, language, attention, and executive function, between pre-SVCI patients and healthy controls. We hypothesized that WM integrity damage and cognitive decline already exist in pre-SVCI patients.

## Materials and Methods

### Study Design and Participants

This case–control study, with prospective recruitment of pre-SVCI cases and a healthy control group, was performed between January 2016 and January 2018 in the Department of Neurology in the China–Japan Friendship Hospital. Vascular risk factors including hypertension (HT), hypercholesterolemia (HC), coronary atherosclerotic disease (CAD), diabetes mellitus (DM), and smoking and alcohol history were collected from all patients.

The patients with pre-SVCI (case group) were defined as having SIVD on MRI with normal global cognitive measures. Two different radiologists assessed the anatomical MRI scans which contained T1-weighted, T2-weighted, fluid-attenuated inversion recovery (FLAIR) and gave nearly the same reports. The patient will be excluded to whom the two radiologists give the different reports. The SIVD patients met the following brain imaging criteria of SIVD (Román et al., 2002): (1) Binswanger-type WM lesions: hyperintensities extending into the periventricular and deep WM, extending caps (>10 mm as measured parallel to the ventricle) or irregular halos (>10 mm with broad, irregular margins and extending into deep WM), and diffusely confluent hyperintensities (>25 mm, irregular shape) or extensive WM changes (diffuse hyperintensity without focal lesions); (2) lacunar cases: multiple lacunas (>2) in the deep gray matter and at least moderate white-matter lesions; and (3) absence of hemorrhages and cortical and/or territorial infarcts and watershed infarcts, signs of normal-pressure hydrocephalus, and specific causes of white-matter lesions. In addition, the visual Fazekas scale was used on FLAIR images to rate the severity of WM hyperintensities (WMHs) into mild (grade 1), moderate (grade 2), and severe (grade 3) WMHs (Fazekas et al., 1987).

The inclusion criteria for the pre-SVCI included (1) literate Han Chinese, education ≥ 6 years, and aged 50–80 years; (2) met with the brain imaging criteria of SIVD above (Román et al., 2002); (3) no cognitive complaints; (4) no impairments of daily life activities with clinical dementia rating (CDR) = 0.5 (Hughes et al., 1982); activities of daily living (ADL) < 23 (Salloway et al., 2004); and (5) normal cognitive screening assessments with MMSE > 26, and MoCA-Beijing version score ≥ 26 (Folstein et al., 1975; Petersen, 2004).

The healthy controls were defined as persons with no neurological and psychiatric disorders, no abnormal findings on conventional brain MRI (brain anatomical MRI was reported normal by the same two radiologists who assessed the MRI for the pre-SVCI group) (Carey et al., 2008; Liu et al., 2019a,b), and no cognitive complaints. For each case, one control was matched by age (within 2 years), sex, and years of education. All enrolled subjects underwent a clinical interview, neurologic examinations, comprehensive neuropsychological assessments, and MRI scanning.

Subjects who met the following criteria were excluded: (1) no completion of neuropsychological testing; (2) Hamilton depression scale score > 17, or anxiety; (3) new strokes within 3 months before baseline; (4) signs of large vessel disease, such as cortical and/or cortico-subcortical non-lacunar territorial infarcts and watershed infarcts or hemorrhages; and (5) leukoencephalopathy as a result of other causes, such as normal pressure hydrocephalus, multiple sclerosis, brain irradiation, and metabolic diseases.

### Neuropsychological Evaluation

We evaluated the cognition status of the subjects with a modified National Institute of Neurological Disorders and Stroke and Canadian Stroke Network-Canadian Stroke Network protocol (Hachinski et al., 2006; Wong et al., 2013). The following cognitive variables were included in the present analysis: (1) global cognition was measured by the MMSE and MoCA; (2) episodic memory was evaluated by the Auditory Verbal Learning Test (AVLT) and the Rey–Osterrieth Complex Figure Delay Tests (R-O delay); (3) language function was examined by the Boston Naming Test (BNT) and the Category Verbal Fluency Test; (4) visuospatial ability was assessed by the Clock Drawing Test (CDT) and the Rey–Osterrieth Complex Figure Copy Test (R-O copy); (5) executive function was assessed by the Trail Making Tests B (TMT-B) and the Stroop Test C right and time; and (6) attention was evaluated by the Digital Span Test (DST) and the Trail Making Test A (TMT-A). The evaluations of all participants were conducted by the same qualified psychologist, and each evaluation required 90 min.

### MRI Acquisition

The MRI data were acquired on a 3.0T Siemens MAGNETOM Prisma MRI scanner. Participants lay supine with the head snugly fixed by a belt. Foam pads were used to restrict head motion, and earplugs were used to minimize the scanner noise. Subjects were told to relax, keep their eyes closed, and remain awake. T1-weighted, sagittal 3D magnetization prepared rapid gradient echo sequences were acquired and covered the entire brain [192 sagittal slices, repetition time (TR) = 2,300 ms, echo time (TE) = 2.32 ms, slice thickness = 0.90 mm, flip angle = 8°, inversion time = 900 ms]. T2-weighted images (TR = 5000 ms, TE = 105 ms, slice thickness = 3 mm, flip angle = 150°, number of slices = 33) and T2-FLAIR images (TR = 9000 ms, TE = 81 ms, slice thickness = 3 mm, flip angle = 150°, number of slices = 25) were acquired. Two sets of DTI data scans were acquired for every subject and then averaged during the data preprocessing. DTI images covering the whole brain were acquired using a single-shot, twice-refocused, diffusion-weighted echo-planar imaging sequence with the following scan parameters: TR = 8,000 ms; TE = 60 ms; 30 diffusion-weighted directions with a *b* value of 1,000 s/mm^2^, and a single image with a *b* value of 0 s/mm^2^; slice thickness = 2 mm; no interslice gap; 75 axial slices; field of view = 282 mm^2^; and voxel size = 2 mm^3^.

### DTI Image Processing

All of the DTI image preprocessing and analyses described below were implemented using a pipeline tool for diffusion MRI, named “Pipeline for analyzing brain diffusion images” (PANDA) (Cui et al., 2013). A similar procedure was shown in our previous studies (Chen et al., 2015; Zhang et al., 2015). First, the DICOM files of all subjects were converted into NIfTI images using the dcm2nii tool embedded in MRI cron. Second, the brain mask was estimated, which was required for the subsequent processing steps. Third, the non-brain spaces in the raw images were removed, leading to a reduced image size, which reduced memory cost and sped up processing in subsequent steps. Fourth, each diffusion-weighted image (DWI) was coregistered to the b0 image using an affine transformation to correct the eddy-current-induced distortions and slow bulk motion-induced inter-gradient misalignment (Chen et al., 2015). The diffusion gradient directions were adjusted accordingly. Fifth, a voxel-wise calculation of the tensor matrix and the diffusion tensor metrics were yielded for each subject, including FA, MD, DA, and DR.

### Mean Diffusion Metrics by an Atlas-Based Segmentation Strategy

White matter atlases (Mori et al., 2008) (e.g., the ICBM-DTI-81 WM labels atlas and the JHU WM tractography atlas) in the standard space allow for parcelation of the WMs into multiple regions of interest (ROIs), each representing a labeled region in the atlas. In our current study, to investigate the diffusion changes in specific tracts, the ICBM-DTI-81 WM label atlas was used to parcel the WMs into 48 ROIs, and only the 40 ROIs in cerebral regions (we focused on the 40 WM tracts within the cerebrum and did not consider the other 8 ROIs within the cerebellum and brain stem) were used for the analysis. Then, the regional mean diffusion metrics including the FA, the MD, the DA, and the DR (Soares et al., 2013; Amlien and Fjell, 2014) were calculated by averaging the values within each region of the WM atlas.

### Statistical Analysis

We used IBM SPSS Statistics for Windows version 22.0 (IBM Corp., Armonk, NY, United States) for all statistical analyses. We assessed the normality of the data with Shapiro–Wilk tests and Q–Q plots. Independent two-sample t-tests were used to assess between-group differences for quantitative variables. The Pearson Chi-square test and Fisher exact probability test were used to compare proportions for categorical variables. Multiple linear regression analysis was used to evaluate the group differences in neuropsychological assessments and diffusion metrics including FA, MD, DR, and DA of the atlas-based ROIs. Age, gender, education years, and groups are the variables when the multiple linear regression analysis was performed. Pearson’s correlation analysis was used to calculate the correlation between diffusion metrics of atlas-based tracts with significant group effects and behavior performance (age, gender, and education years were included as covariates). For all analyses, a two-tailed *p* value < 0.05 was considered statistically significant. Bonferroni correction was performed in multiple comparisons of 40 atlas-based ROIs (*p* < 0.05/40) and correlation between diffusion metrics of atlas-based tracts with significant group effects and neuropsychological assessments (*p* < 0.05/14).

## Results

### Demographics and Neuropsychological Testing

**FIGURE 1:**
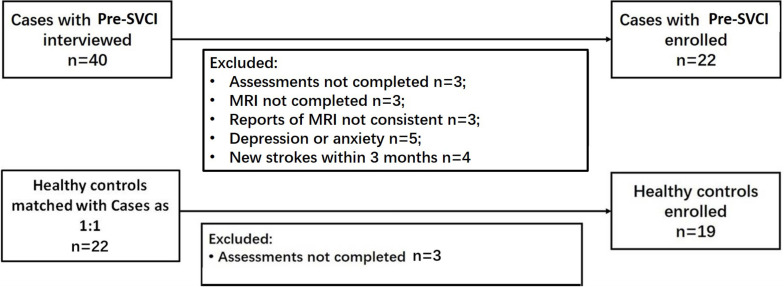
Flowcharts for study enrollment of case participants and control participants. Pre-SVCI is subcortical ischemic vascular disease with normal global cognition measures.

According to the inclusion and exclusion criteria, we ultimately enrolled 22 cases and 19 healthy controls. Figure 1 shows the participant enrollment process. The demographic and clinical characteristics of the participants are presented in Table 1. The distribution of age and education years was normal. There were no significant differences in age, sex, years of education, history of DM, CAD, HC, alcohol intake, and smoking. As expected, there was a significant difference in the history of hypertension between the two groups.

**TABLE 1:** Demographics and clinical characteristics of each group.

	Pre-SVCI (*n* = 22)	H (*n* = 19)	t/X^2^	Sig
Sex (male/female)	10/12	7/12	0.312	0.577
Age (years)	65.14 ± 1.67	61.58 ± 1.80	−1.543	0.131
Education (years)	12.36 ± 0.76	12.68 ± 0.81	0.304	0.763
Smoking (n)	8 (36.4%)	5 (26.3%)	0.475	0.491
Alcohol (n)	2 (9.1%)	0 (0%)	–	0.490
HT (n)	11 (50.0%)	2 (10.5%)	5.627	0.018*
HC (n)	11 (50.0%)	4 (21.1%)	2.540	0.111
CAD (n)	4 (18.2%)	2 (10.5%)	0.062	0.804
DM (n)	8 (36.4%)	5 (26.3%)	0.475	0.491

Values are mean ± SD. The comparisons of years of age and education between the two groups were performed using the *t*-test. The Pearson chi-square was used when expected cell counts are equal to 5 or more. Continuity correction was used when expected cell counts ≥ 1 and < 5. If these conditions are not met, the Fisher exact probability test was be used instead of the chi-square test. *P* < 0.05 was considered significant. *P < 0.05; HT, hypertension; HC, hypercholesterolemia; CAD, coronary atherosclerotic disease; DM, diabetes mellitus; pre-SVCI, subcortical ischemic vascular disease with normal global cognition measures; H, healthy.

Table 2 presents the cognitive assessment results. The pre-SVCI group exhibited significant differences in the MoCA, Rey–Osterrieth Complex Figure (R-O)-copy, and TMT-B test compared with the H group.

**TABLE 2:** Neuropsychological tests result for each group.

	Pre-SVCI (*n* = 22)	H (*n* = 19)	*t*-value	Effect sizes (standardized beta)	*p*-value
**General mental status**					
MMSE MoCA	27.95 ± 1.53 26.55 ± 0.67	28.79 ± 1.36 27.97 ± 0.74	−1.302 −3.984	−0.208 −0.544	0.201 <0.001*
**Episodic memory**					
AVLT-delay	7.73 ± 5.86	6.95 ± 2.46	0.132	0.022	0.895
AVLT-total	32.55 ± 7.42	33.79 ± 6.75	−0.444	−0.076	0.660
R-O delay	18.23 ± 6.89	17.47 ± 7.71	−0.696	−0.115	0.491
**Spatial processing**					
R-O copy	34.55 ± 2.24	35.37 ± 0.96	−2.151	−0.405	0.045*
CDT	24.00 ± 6.44	24.32 ± 6.77	0.176	0.030	0.861
**Executive function**					
Stroop C-time	98.57 ± 10.35	78.82 ± 11.40	1.982	0.311	0.055
Stroop C-right	47.80 ± 2.26	45.31 ± 2.49	1.186	0.194	0.243
TMT b	163.57 ± 15.56	113.71 ± 17.16	5.746	0.700	<0.001*
**Language ability**					
BNT	26.68 ± 1.89	25.79 ± 2.02	1.302	0.213	0.201
CVFT	42.00 ± 12.98	49.58 ± 9.36	−1.946	−0.312	0.059
**Attention**					
SDMT	34.77 ± 17.08	39.37 ± 7.96	−0.633	−0.099	0.531
TMTa	51.82 ± 31.76	51.84 ± 12.23	−0.087	−0.015	0.931

The differences in neuropsychological scores between the two groups were tested for significance with multiple linear regression analysis adjusted for age, sex, and education. *P* < 0.05 was considered significant. *P < 0.05; MMSE, Mini-Mental State Examination; MoCA, Montreal cognitive assessment; AVLT, Auditory Verbal Learning Test; R-O delay, Rey–Osterrieth Complex Figure delay tests; R-O copy, Rey–Osterrieth Complex Figure copy test; CDT, Clock drawing task; BNT, Boston Naming Test; CVFT, Category Verbal Fluency Test; SDMT, Symbol Digit Modalities Test; TMT, Trail Making Test; Clock Drawing Test; pre-SVCI, subcortical ischemic vascular disease with normal global cognition measures; H, healthy.

### Results of the Atlas-Based Tract ROIs

**FIGURE 2:**
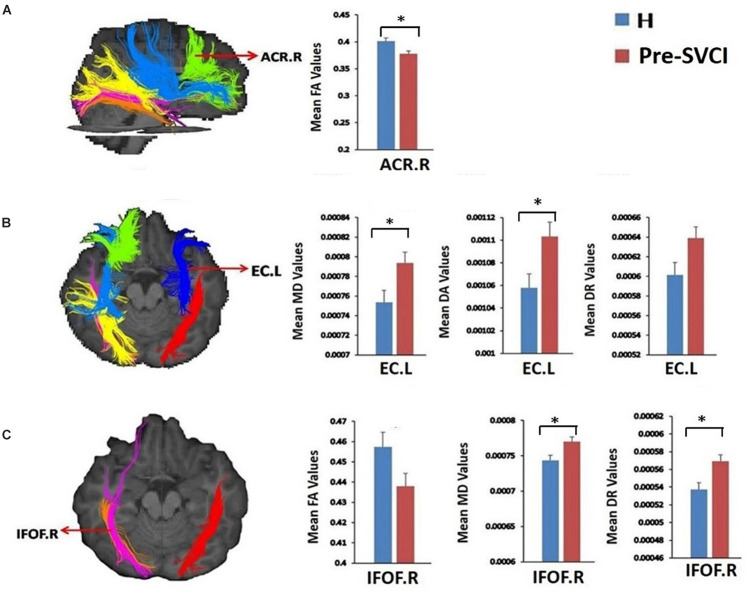
The tracts where diffusion metrics remained significant between the two groups after Bonferroni correction for multiple comparisons. **(A)** Mean FA values of ACR.R; **(B)** Mean MD, DA, and DR values of EC.L; **(C)**, mean FA, MD, and DR values of IFOF.R. *is significant at *p* < 0.05/40 after Bonferroni correction. FA, fractional anisotropy, MD, mean diffusivity; DA, axial diffusivity; DR, radial diffusivity; pre-SVCI, subcortical ischemic vascular disease with normal global cognition measures; H, healthy; L, left; R, right; ACR, anterior corona radiata; EC, external capsule; IFOF, inferior fronto-occipital fasciculus.

Table 3 shows the diffusion metrics of WM tracts which are significantly different when comparing the pre-SVCI group to the H group. Compared with the control group, the pre-SVCI group exhibited significantly decreased FA in the right anterior corona radiata (ACR) and inferior fronto-occipital fasciculus (IFOF) (*p* < 0.05). Meanwhile, increased MD in the right side of the posterior thalamic radiation (PTR), the inferior longitudinal fasciculus (ILF), the IFOF, and the left side of the external capsule (EC) were observed (*p* < 0.05). The case group also exhibited increased DA on the bilateral side of the ILF, the EC, and the right side of the PTR and increased DR in the left EC and the right IFOF (*p* < 0.05). Among them, FA values of right ACR, MD values of left EC and right IFOF, DA values of left EC, and DR values of right IFOF are still significantly different after Bonferroni correction (*p* < 0.05/40) for the multiple comparisons (Figure 2).

**TABLE 3:** Group comparisons of mean DTI diffusion metrics of white matter tracts in two groups.

	Pre-SVCI (*n* = 22)	H (*n* = 19)	*t*-value	Effect sizes (standardized beta)	*p*-value
**ACR.R**					
FA	0.3778 ± 0.0213	0.4013 ± 0.0213	−4.150	−0.566	<0.001*
**ILF.L**					
DA (10^–3^)	1.290 ± 0.495	1.250 ± 0.260	2.549	0.402	0.015
**EC.R**					
DA (10^–3^)	1.084 ± 0.415	1.046 ± 0.198	3.269	0.471	0.002
**PTR.R**					
MD (10^–4^)	8.025 ± 4.056	7.749 ± 3.484	2.885	0.436	0.007
DA (10^–3^)	1.329 ± 0.441	1.297 ± 0.443	2.352	0.367	0.024
**ILF.R**					
MD (10^–4^)	8.129 ± 3.673	7.887 ± 2.014	2.294	0.361	0.028
DA (10^–3^)	1.277 ± 0.438	1.250 ± 0.261	2.307	0.366	0.027
**EC.L**					
MD (10^–4^)	7.938 ± 3.569	7.537 ± 2.232	4.331	0.586	<0.001*
DA(10^–3^)	1.103 ± 0.457	1.058 ± 0.261	3.893	0.550	<0.001*
DR(10^–4^)	6.391 ± 0.366	6.016 ± 0.247	3.399	0.505	0.002
**IFOF.R**					
FA	0.43795 ± 0.0219	0.4575 ± 0.0301	−2.337	−0.369	0.025
MD (10^–4^)	7.703 ± 2.302	7.436 ± 1.519	3.841	0.540	<0.001*
DR(10^–4^)	5.690 ± 0.265	5.371 ± 0.226	3.780	0.538	<0.001*

The differences in diffusion metrics (i.e., FA, MD, DA, and DR) of white matter tracts between the two groups were tested for significance with multiple linear regression analysis adjusted for age, sex, and education. *P* < 0.05 was considered significant. * is significant at *p* < 0.05/40 after Bonferroni correction; FA, fractional anisotropy; MD, mean diffusivity; DA, axial diffusivity; DR, radial diffusivity; pre-SVCI, subcortical ischemic vascular disease with normal global cognition measures; H, healthy. L, left; R, right. For the abbreviations of WM tracts, see Supplementary Table 1.

### Correlations Between ROI-Wise Diffusion Metrics and Behaviors

**FIGURE 3:**
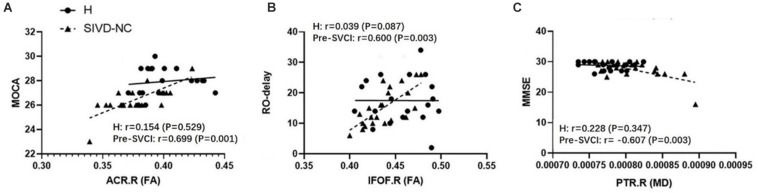
The significant correlations between ROI-wise diffusion metrics and behaviors in the pre-SVCI group and the H group after Bonferroni correction for multiple comparisons. **(A)** MoCA. **(B)** R-O delay. **(C)** MMSE. MoCA, Montreal cognitive assessment; R-O delay, Rey–Osterrieth Complex Figure delay tests; MMSE, Mini-Mental State Examination; FA, fractional anisotropy, MD, mean diffusivity; pre-SVCI, subcortical ischemic vascular disease with normal global cognition measures; H, healthy; L, left; R, right; ACR, anterior corona radiata; IFOF, inferior fronto-occipital fasciculus; PTR, posterior thalamic radiation.

We examined the relationship between the mean values of the diffusion metrics of the ROIs extracted from the significant WM regions and neuropsychological scores of the pre-SVCI group and healthy controls. In the pre-SVCI group, the mean FA values of the right ACR were significantly correlated with the MoCA (*r* = 0.699, *p* = 0.001). The R-O delay was positively correlated with the mean FA values (*r* = 0.600, *p* = 0.003) but negatively correlated with the MD values (*r* = −0.441, *p* = 0.040) of the right IFOF. Negative correlations were observed between the mean MD value of the left EC and the Category Verbal Fluency Test (CVFT) (animal) (*r* = −0.564, *p* = 0.006). Negative correlations can be seen between the mean MD value of the right PTR and the Stroop C-right and MMSE scores (*r* = −0.458, *p* = 0.032 and *r* = −0.607, *p* = 0.003, respectively). Notably, the correlations between the FA of right ACR and MoCA, the FA of right IFOF and RO-delay, and the MD of right PTR and MMSE were still significant after Bonferroni correction (*p* < 0.05/14) for multiple comparison (Figure 3).

In the control group, there was no negative or positive correlation between the diffusion metrics of the significant WM tracts and neuropsychological scores.

## Discussion

In the current study, we evaluated WM alterations and cognitive declines in the early stage of SVCI (pre-SVCI), compared with a well-matched healthy group. We made several observations. (1) Executive function and spatial processing were already declined in the pre-SVCI patients. (2) Some long associative and projective bundles were damaged in the pre-SVCI group. In particular, the right IFOF was found to have decreased FA, increased MD, and increased DR. (3) The mean values of the diffusion indices in some specific WM tracts in the pre-SVCI group were significantly correlated with neuropsychological assessments that related to executive functions or spatial processing.

According to the brain connectome theory, disruptions in the global connections between cortical and subcortical networks are partially related to damage in WM fibers (Lo et al., 2010). Thus, SVCI which is characterized by extensive cerebral WM lesions in the periventricular/deep cerebral WM (Román et al., 2002; Rosenberg et al., 2014; Tu et al., 2017), tends to result in cognitive impairment. Previous studies (Biesbroek et al., 2017; Tu et al., 2017; Liu et al., 2019b) have revealed that SVCI patients exhibit nearly global changes with decreased FA and increased MD in WM tracts especially those located in thalamic-prefrontal and caudate-prefrontal pathways. It has been proven that these two pathways are markedly related to neuropsychological assessments of executive function, attention, and processing speed in many task-related functional studies (Block et al., 2007; Müller-Oehring et al., 2015; Liu et al., 2018). Our study showed that the TMT-B assessment was significantly different between the patients and healthy controls. However, the above studies mainly focus on patients at the stages of SvMCI or SVaD and neglect the asymptomatic stage of disease when individuals suffer vascular damage without cognitive impairment.

Our study found that some types of long associative and projective WM such as right ACR, IFOF, and left EC were extensively damaged in the pre-SVCI group even given the strictest Bonferroni correction for multiple comparisons. However, Liu et al. (2019b) found that approximately all cerebral WMs were symmetrically involved in the pre-SVCI group but were less distinct than those in the SVCI group. Our different result from the previous study was mainly due to the discrepant inclusion criteria for including the pre-SVCI group. We used two screening scales, i.e., the MMSE and MoCA, both to include patients with normal global measures. The MoCA is considered more sensitive for screening VCI, which surpasses the well-known limitations of the MMSE (Dong et al., 2010). Because of the lack of sensitivity in detecting subtle cognitive changes, particularly visuospatial and executive function impairments, using the MMSE alone may lead to a false-negative bias in the recruitment of patients. In our study, the patients had a significant difference compared with the healthy controls in MoCA, but not the MMSE, further confirming the above view.

Our results demonstrated that IFOF might be one kind of WM tracts that are much more easily demyelinated in vascular disease. Notably, three of the DTI-derived indices changed with decreased FA, increased MD, and increased DR in the right IFOF. IFOF is one of the longest major associative bundles that was recognized and depicted in 2007 (Schmahmann and Pandya, 2007). It connects the occipital cortex, the superior parietal lobe, and the temporo-basal areas to the frontal cortex (Martino et al., 2010). Some DTI studies have shown that the IFOF is a probable crucial tract in reading, attention, and visual processing, especially the right IFOF in spatial attention and neglect (Catani and Thiebaut de Schotten, 2008; Urbanski et al., 2008, 2011). Consistently, based on the correlation analysis between WM impairment and cognitive decline, we found that the RO-delay, which represents long-term memory and visuospatial function, was positively correlated with the FA value of the right IFOF after the strictest Bonferroni correction, which further identified that the damage of IFOF is correlated with the decline of visual processing. The right IFOF may be impaired at the early stage because of its long course, which can easily result in myelin injury. According to our findings, the DR of IFOF.R was significantly increased. An increasing DR reflects a decline in myelin sheath integrity (Soares et al., 2013). Thus, we can further infer that long tracts, such as the IFOF, which course from the front regions to the end of the brain, are probably predamaged before the prefrontal thalamus circus. Furthermore, we found that the significantly damaged tracts in the pre-SVCI patients were mainly concentrated in the right hemisphere. The integrity of IFOF, PTR, and ATR in the right hemisphere was extensively damaged which were also correlated with the declines in visuospatial and executive functions. So far, studies (Kleinman et al., 2007; Giussani et al., 2010; Kontaxopoulou et al., 2017; Vilasboas et al., 2017; Bernard et al., 2018) have increasingly found that the non-dominant right hemisphere is responsible for primary cognitive functions such as visuospatial, intentional process, and social cognition. This is consistent with the findings in our study. But why the WMs in right hemisphere are damaged earlier than left hemisphere in SIVD patients is not clear.

There are several limitations in this study. First, although according to previous studies, we supposed that SIVD patients with normal global cognitive measures could be pre-SVCI patients, we could not clearly determine whether these pre-SVCI patients will develop into SVCI, remain unchanged, or improve as a consequence of brain plasticity or reserve capacity. This bias leads our results to underestimate the difference between the cases and the controls. Besides, as previous studies, we did not report measures of head motion for each group and just rely on registration-based correction methods that cannot eliminate the full effects of head motion on the DW images (Yendiki et al., 2014). Therefore, longitudinal follow-up studies with a large-scale and more accurate and comprehensive methods to correct head motion artifacts are needed in the future. Second, our study focused on only WM alterations, but whether cerebral blood perfusion or other elements are correlated with cognitive decline still needs further exploration. Moreover, we did not assess the degree of gray matter atrophy, which can have an impact on cognitive functions. Third, this study involved Chinese individuals, and other ethnic groups need to be further studied.

## Conclusion

In summary, our study indicated that in SIVD patients, even with normal global cognitive measures, some types of long course tracts, especially the tracts in the right hemisphere, were damaged. Furthermore, damage to these tracts was associated with a decline in some specific cognitive domains, such as executive functions and spatial processing domains. These results indicated that pre-SVCI patients are likely at an ultra-early stage of SVCI, and there was a very high risk of this condition becoming SVCI. To prevent the progression of SVCI, longitudinal studies are needed to explore the dynamic changes from the early stage to the clinical stage of SVCI and to evaluate the value of DTI in predicting the process of SVCI.

## Data Availability Statement

The raw data supporting the conclusions of this article will be made available by the authors, without undue reservation.

## Ethics Statement

The studies involving human participants were reviewed and approved by the Institutional Review Board of China-Japan Friendship Hospital. The patients/participants provided their written informed consent to participate in this study. Written informed consent was obtained from the individual(s) for the publication of any potentially identifiable images or data included in this article.

## Author Contributions

YQ and DP: conceptualization. XH and JZ: methodology. YQ, XH, YL, and WS: formal analysis and investigation. YQ: writing – original draft preparation. SZ: writing, review, and editing. ZZ and DP: supervision. All authors contributed to the article and approved the submitted version.

## Conflict of Interest

The authors declare that the research was conducted in the absence of any commercial or financial relationships that could be construed as a potential conflict of interest.

## Publisher’s Note

All claims expressed in this article are solely those of the authors and do not necessarily represent those of their affiliated organizations, or those of the publisher, the editors and the reviewers. Any product that may be evaluated in this article, or claim that may be made by its manufacturer, is not guaranteed or endorsed by the publisher.
