# Supplementary material for: The Associations Between White Matter Disruptions and Cognitive Decline at the Early Stage of Subcortical Vascular Cognitive Impairment: A Case–Control Study
Source: Front Aging Neurosci. 2021 Aug 2;13:681208. doi: 10.3389/fnagi.2021.681208 (PMC8364958; doi:10.3389/fnagi.2021.681208)
Supplement: Supplementary file 1 [file Table_1.DOCX]

**Supplementary table 1.** The 40 WM tract ROIs based on the ICBM-DTI-81 WM labels atlas within cerebral regions

| Index | Abbr. | Tracts | Index | Abbr. | Tracts |
| --- | --- | --- | --- | --- | --- |
| 1 | gCC | Genu of corpus callosum | 21 | ILF.R | Inferior longitidinal fasciculus.R |
| 2 | bCC | Body of corpus callosum | 22 | ILF.L | Inferior longitidinal fasciculus.L |
| 3 | sCC | Splenium of corpus callosum | 23 | EC.R | External capsule.R |
| 4 | cbFN | Fornix (column and body of fornix) | 24 | EC.L | External capsule.L |
| 5 | CP.R | Cerebral peduncle.R | 25 | CCG.R | Cingulum (cingulate gyrus).R |
| 6 | CP.L | Cerebral peduncle.L | 26 | CCG.L | Cingulum (cingulate gyrus).L |
| 7 | ALIC.R | Anterior limb of internal capsule.R | 27 | CH.R | Cingulum.(hippocampus).R |
| 8 | ALIC.L | Anterior limb of internal capsule.L | 28 | CH.L | Cingulum.(hippocampus).L |
| 9 | PLIC.R | Posterior limb of internal capsule.R | 29 | F/ST.R | Fornix (cres) / Stria terminalis (can not be resolved with current resolution).R |
| 10 | PLIC.L | Posterior limb of internal capsule.L | 30 | F/ST.L | Fornix (cres) / Stria terminalis (can not be resolved with current resolution).L |
| 11 | RIC.R | Retrolenticular part of internal capsule.R | 31 | SLF.R | Superior longitudinal fasciculus.R |
| 12 | RIC.L | Retrolenticular part of internal capsule.L | 32 | SLF.L | Superior longitudinal fasciculus.L |
| 13 | ACR.R | Anterior corona radiata.R | 33 | SFOF.R | Superior fronto-occipital fasciculus (could be a part of anterior internal capsule).R |
| 14 | ACR.L | Anterior corona radiata.L | 34 | SFOF.L | Superior fronto-occipital fasciculus (could be a part of anterior internal capsule).L |
| 15 | SCR.R | Superior corona radiata.R | 35 | IFOF.R | Inferior.fronto-occipital.fasciculus.R |
| 16 | SCR.L | Superior corona radiata.L | 36 | IFOF.L | Inferior.fronto-occipital.fasciculus.L |
| 17 | PCR.R | Posterior corona radiata.R | 37 | UF.R | Uncinate fasciculus.R |
| 18 | PCR.L | Posterior corona radiata.L | 38 | UF.L | Uncinate fasciculus.L |
| 19 | PTR.R | Posterior thalamic radiation (include optic radiation).R | 39 | TAP.R | Tapetum.R |
| 20 | PTR.L | Posterior thalamic radiation (include optic radiation).L | 40 | TAP.L | Tapetum.L |
